# Acute acalculous cholecystitis determining Mirizzi syndrome: case report and literature review

**DOI:** 10.1186/1471-2482-14-90

**Published:** 2014-11-15

**Authors:** Marco Milone, Mario Musella, Paola Maietta, Dario Guadioso, Anna Pisapia, Guido Coretti, Giovanni De Palma, Francesco Milone

**Affiliations:** University of Naples “Federico II”, Via Pansini 5, Naples, 80131 Italy

**Keywords:** Acute cholecystitis, Mirizzi, Acalcholus, Jaundice, Stone

## Abstract

**Background:**

Although Mirizzi syndrome is widely reported in literature, little is known about acute acalcholous cholecystitis determinig the findings of a Mirizzi syndrome.

**Case presentation:**

We report a case of MRCP-confirmed Mirizzi syndrome in acute acalculous cholecystitis resolved by surgery.

**Conclusion:**

Acute acalcholosus cholecystitis determinig a Mirizzi Syndrome should be included in the Mirizzi classification as a type 1. Thus it could be useful to divide the type 1 in two entity (compression by stone and compression by enlarged gallbladder). Magnetic Resonance should be considered the preferred diagnostic tool in any case of Mirizzi syndrome suspicious.

## Background

Mirizzi syndrome (MS) is a relatively rare complication of gallstone disease. Surgery is the most effective treatment of MS, but it is accompanied with an increased risk of bile duct injury. Severe inflammation and adhesions in the subhepatic area are very common and almost always involve the hepatoduodenal ligament and they distort the normal anatomic relationships and proportions. Acute acalculous cholecystitis (AAC) can lead to the development of a condition which is very similar to MS in its clinical course and imaging findings. We were able to identify up to now only four well-documented cases in the literature [1–4]. Although Mirizzi syndrome is widely reported in literature, little is known about acute acalcholous cholecystitis determinig the finding of a MS.

## Case presentation

**Figure 1:**
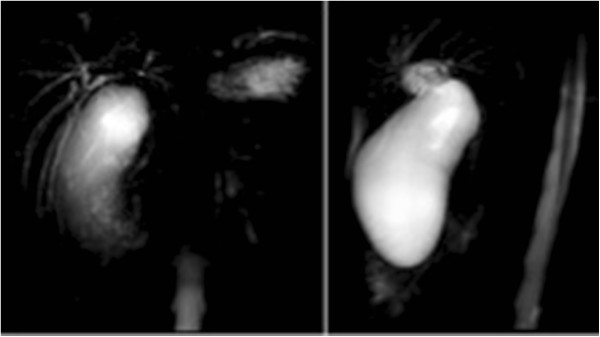
**MRCP demonstrates gallbladder infundibulum applying a “mass effect” to hepatic hilum.**

A 25 years old woman (body mass index, BMI 22Kg/m^2^), affected by Beckwith-Wiedemann Syndrome (an overgrowth disorder usually present at birth, characterized by an increased risk of childhood cancer and certain congenital features) was admitted urgently with severe pain in the right hypochondrium, acholic stools, hyperchromic urine, nausea, vomiting and fever. Her past medical history was unremarkable. Physical examination revealed jaundice and tenderness in the right hypochondrium with localized rebound and guarding, positive Murphy's sign. Furthermore a distended gallbladder was appreciable. Total bilirubin was 5,44 mg/dl, aspartate aminotransferase 83 IU/ml, alanine aminotransferase 332 IU/ml, alkaline phosphatase 244 IU/ml. The white blood cell count was 7.300/ml. Ultrasonography showed a distended and hydropic gallbladder measuring 14 cm in its longitudinal axis with moderate wall thickening and marked intrahepatic biliary ductal dilatation. No gallstones were seen. Upper abdominal MRI scan (FFE T1 “in and out phase”, TSE T2 and T2 Spir axial scans; TSE T2 Bh coronal scans; cholangiographic FFE T2 radial scans) demonstrated distended gallbladder with homogeneus contents, mild perichepatic and pericholecistic fluid, severe and widespread dilatation of intrahepatic ducts without dark filling defects suggesting biliary lithiasis (Figure 1). Furthermore MRI showed gallbladder infundibulum applying a “mass effect” to hepatic hilum. Thus a laparotomic cholecystectomy was uneventfully performed. During surgery a massively dilated, edematous gallbladder very close to the hepatic hilum was found. Pathologic examination of the gallbladder showed evidence of severe acute inflammation and epithelial necrosis but no stones in the gallbladder or cystic duct. No carcinoma was evident. She was discharged after 5 days with a complete recovery. Antiobiotcs were administered for three days after surgery. Postoperative course was uneventful during the three months follow-up.

Written informed consent was obtained from the patient for publication of this case report and any accompanying images. A copy of the written consent is available for review by the Editor of this journal.

## Discussion

Mirizzi syndrome (MS) is an uncommon complication of gallstone disease, with a reported incidence between 0.06% and 5.7% in patients undergoing cholecystectomy [5–7]. The condition was originally described by Kehr in 1905 [5, 8], but it was named after Pablo Mirizzi several years later, who defined the syndrome as a benign common hepatic duct obstruction due to gallstone impaction in the gallbladder neck resulting in local inflammation and bile duct spasm [5, 9]. As it was eventually recognized that there is no physiological sphincter of the hepatic duct, MS was finally attributed to extrinsic compression of the common hepatic duct by gallstones impacted in the cystic duct or the gallbladder neck [5, 10]. Bile duct wall necrosis and subsequent cholecystobiliary fistula caused by chronic inflammation is a rare sequence of the disease.

Mirizzi described included some essential pathophysiological elements: a long cystic duct; parallel to the bile duct and a low insertion of the cystic duct into the bile duct; single large or multiple small gallstones impacted in the Hartmann's pouch or in the gallbladder infundibulum and cystic duct; obstruction of the common hepatic duct secondary to external compression by the impacted stone; jaundice [9, 11–15].

It is of particular importance to surgeons because the diagnosis may not be appreciated preoperatively and because the surgical treatment of this condition is associated with a significantly increased risk of bile duct injury [16]. Accurate definition of the biliary anatomy preoperatively, when possible, is thus critical for optimal surgical planning [16]. AAC can be complicated with extrinsic compression of the common hepatic/common bile duct by the enlarged and inflamed gallbladder, which was followed by jaundice. Its mechanism is very similar to MS, when the bile duct is compressed from outside due to a stone impacted at the gallbladder neck or cystic duct [16].

We have identified only four documented similar cases reported in the literature [1–4]. In three of these cases, compression of the common hepatic/common bile duct by the inflamed gallbladder was confirmed by endoscopic retrograde cholangiopancreatography and patients were subjected to surgery. Just in one case the diagnosis of compression of bile duct by the inflamed and enlarged gallbladder was confirmed by MRCP rather than ERCP [4]. But in this same case surgery was avoided and patient was subjected to a conservative therapy. So we can affirm this is the first reported case of MRCP-confirmed Mirizzi syndrome in acute acalculous cholecystitis resolved by the surgery. At last our case can be considered as unique because of compression of hepatic hilum rather than common hepatic/common bile duct by distended gallbladder.

It is interesting to note that the terminology to describe this condition has not been agreed upon [4]. In Ippolito's paper [1] he called the condition “Acute acalculous cholecystitis associated with common hepatic duct obstruction: a variant of Mirizzi's syndrome”. K. Mergener et al. [2] entitled their article “Pseudo-Mirizzi syndrome in acute cholecystitis”, while S. Ahlawat [16] used the title – “Acute acalculous cholecystitis simulating Mirizzi syndrome: a very rare condition”. Finally YN Shiryajev et al. [4] in their work speaked about “Acute acalculous cholecystitis complicated by MRCP-confirmed Mirizzi syndrome: A case report”.

The most widely accepted classification of Mirizzi Syndrome was proposed by McSherry et al. [17], who described two types: type I includes partial or complete obstruction of the common hepatic duct due to external compression; type II refers to the formation of a communication between the gallbladder neck or the cystic duct and the common hepatic duct. Csendes et al. further subclassified cholecystobiliary communication into three types according to the diameter of the biliary fistula [18]. In this classification, type II is a cholecystobiliary fistula that involves less than one-third of the circumference of the bile duct, type III is a fistula that involves up to two-thirds of the bile duct circumference, and type IV is a fistula with complete bile duct destruction [5]. According to Shiryajev [4] we believe that the above-mentioned entity can be considered to be a special kind of MS, but we think that this condition should be considered a variation of the MS type 1. Furthermore Mirizzi's syndrome is not easily diagnosed in the preoperative period. Because of these limitations (the syndrome is not associated with a well-defined set of demographics or unique clinical features), the preoperative diagnosis of Mirizzi's syndrome depends heavily on appropriate imaging studies [24]. Ultrasonography is usually the initial radiological investigation in case of obstructive jaundice. Further radiological investigation are CT or MRI. At last patients can be referred to ERCP (important not only for diagnosis but also as part of the treatment of some cases of Mirizzi syndrome) and/or surgical procedures. Typical cholangioresonance or magnetic resonance cholangiopancreatography (MRCP) findings of Mirizzi syndrome [19, 20] include an impacted stone in the gallbladder neck, estrinsic compression of the common hepatic duct, dilatation of the intrahepatic and common hepatic ducts above the level of impaction with a normal choledochus, a contracted gallbladder with wall-thickening. Moreover MRI is useful to exclude, by additional sequences, other causes of bile tract obstruction.

Recent case reports suggest that magnetic resonance cholangiography can be an effective method of diagnosing MS using as criteria [21]: (1) dilatation of the biliary tree above the level of the gallbladder neck, (2) impaction of a stone in the gallbladder neck, and (3) a normal caliber CBD below the level of impaction [6, 16].

The diagnosis of MS in acalculous cholecystitis was confirmed by MRCP in our case as well as in the case reported by Shiryajev [4]. Thus, although further studies are needed to give definitive conclusion, we think that MR should be an effective way to diagnose any kind of MS including this rare condition of acute acalcholosus cholecystitis determinig a Mirizzi syndrome.

## Conclusion

Acute acalculous cholecystitis may rarely present as a Mirizzi syndrome, which might be considered a dimension of the type I MS classification. Thus it could be useful to divide the MS type 1 in two entity (compression by stone and compression by enlarged gallbladder). MR should be considered the preferred diagnostic tool in any case of MS suspicious.

Open cholecystectomy may be the preferred therapeutic option, whereas laparoscopic treatment may be attempted, if laparoscopic expertise is available.
