# Correction to: Acute acalculous cholecystitis determining Mirizzi syndrome: case report and literature review

**DOI:** 10.1186/s12893-018-0404-5

**Published:** 2018-08-30

**Authors:** Marco Milone, Mario Musella, Paola Maietta, Dario Gaudioso, Anna Pisapia, Guido Coretti, Giovanni De Palma, Francesco Milone

**Affiliations:** University of Naples “Federico II”, Via Pansini 5, 80131 Naples, Italy

## Correction

Following publication of the original article [1], the authors reported that one of the authors’ names is spelled incorrectly. In this Correction the incorrect and correct author name are shown.

Originally the author name has been published as:

- Dario Guadioso

The correct author name is:

- Dario Gaudioso
